# Review of the *Eospilarctia yuennanica* group (Lepidoptera, Erebidae, Arctiinae) from the Indo – Himalayan region, with description of two new species and one subspecies

**DOI:** 10.3897/zookeys.204.3056

**Published:** 2012-06-25

**Authors:** Aidas Saldaitis, Povilas Ivinskis, Thomas Witt, Oleg Pekarsky

**Affiliations:** 1 Nature Research Centre, Akademijos str. 2, LT–08412 Vilnius-21, Lithuania; 2 Nature Research Centre, Akademijos str. 2, LT–08412 Vilnius-21, Lithuania; 3 Museum Witt München, Tengstrasse 33, D–80796 München, Germany; 4 Felsőerdősor u. 16-18, H-1068 Budapest, Hungary

**Keywords:** Arctiinae, new species, new subspecies, China, Myanmar, Vietnam

## Abstract

Twonew *Eospilarctia* species and one new subspecies from China, Myanmar and Vietnam, respectively, are described. Superficially the new species *Eospilarctia maciai* **sp. n.**,* Eospilarctia naumanni* **sp. n.** and *Eospilarctia yuennanica fansipana* **ssp. n.** resemble related congeners but they can be distinguished by the differences in wing pattern, genitalia and distribution provided. *Eospilarctia yuennanica guangdonga* Dubatolov, Kishida & Wang, 2008 is upgraded to species level. A checklist of the genus *Eospilarctia* and a key to the *Eospilarctia yuennanica* (Daniel, 1943) species-group, based on external characters and male genitalia, is presented.

## Introduction

The genus *Eospilarctia* Kôda, 1988 contains 13 species and two subspecies mainly distributed in China (ten species), Taiwan, Japan and Vietnam. All species of genus *Eospilarctia* have typical wings patterns concist from longitudinal white or yellowish streaks. Identification of species is available by compare genital structures of similar species. Comparisons of the genitalic structures of specimens found in the Zoologische Staatssammlung der Bayerischen Staaten (Munich, Germany)/Museum of Thomas Witt with *Eospilarctia yuennanica* –cotypeled to the description of the new species and subspecies.

## Materials and methods

We examined specimens of *Eospilarctia* Kôda preserved in the Zoologische Staatssammlung der Bayerischen Staaten, Munich, Germany (ZSM) and the Museum of Thomas Witt (MWM). Examined specimens were collected in China, Myanmar and Vietnam using ultraviolet light traps. 31 genital slides were prepared and 124 photos were made. Reference to relevant literature (Freina de 1999, Fang 2000, Dubatolov, Kishida and Wang 2008; Dubatolov 2010) and consultation with expert taxonomists in addition to numerous genitalia dissections were used to resolve the taxonomy of *Eospilarctia yuennanica* species group.

Examination of morphology: after maceration, male and female genitalia were dissected and mounted in euparal on glass side. The abdominal integument was cut lengthwise, descaled, and mounted on the slide, dissection of genitalia follows Lafontaine (2004). Photos of genitalia where made using microscope Nikon SMZ745T and camera Moticam 2500.

## Systematic accounts

### Genus Eospilarctia

Kôda, 1988

http://species-id.net/wiki/Eospilarctia

Eospilarctia Kôda, 1988. Tyô to ga Zoologist 39(1): 39–45

#### Type species:

*Seiarctia lewisii* Butler, 1885, by original designation.

#### Genus characteristic.

Species of this genus typically have a medium wingspan, long bipectinate antennae, a red patagium, a narrow red abdomen, yellow tegulae, white or yellow longitudinal fascia transversing brown forewings, pale white or yellow-white hindwings with various sized brown spots in terminal and discal areas.

**Male genitalia.** Uncus massive, wide base abruptly narrowing at the tip; tegumen medium size; gnathos reduced; valvae simple with even edges but concave, rough or rounded at the apex; juxta wide with wide excision; saccus short, semioval form; aedeagus straight, longer than valvae; vesica multiplex.

**Female genitalia.** Papilla analis massive; apophysis posterioris approximately 2.5 times shorter than apophysis anterioris; ductus short; bursa bisaccate, rounded, without signum.

#### Distribution.

Fourteen species of *Eospilarctia* are distributed in China, Myanmar, Vietnam, Taiwan and Japan.

#### Key to *Eospilarctia* species related to *Eospilarctia yuennanica yuennanica* (Daniel, 1943) based on external characters

**Table:** 

1	Forewings costal streak wide (Plate 1: fig. 3)	2
–	Forewings costal streak narrow (Plate 1: figs 1, 2, 4–6)	3
2	Forewings costal streak extending from base to apical streak; postmedian streak short directed to costa (Dubatolov et al. 2008, fig. 7, p. 134)	*Eospilarctia guangdonga* (China: Guangdong)
–	Forewings costal streak extending from base to beginning, base of radial vein; postmedian streak short directed to termen (Plate 1: fig. 3)	*Eospilarctia yuennanica fansipana* ssp. n. (Vietnam: Fan-si-pan)
3	Forewings pale light brown	*Eospilarctia yuennanica yuennanica* (China: Sichuan)
–	Forewings highly contrasted (Plate 1: figs 4, 5)	4
4	Forewings costal streak extending from base to apical streak (Plate 1: fig. 5)	*Eospilarctia naumanni* sp. n. (Myanmar: Kachin)
–	Forewings costal streak extending from base to beginning, base of radial vein (Plate 1: fig. 4)	*Eospilarctia maciai* sp. n. (China: Yunnan)

#### Key to species *Eospilarctia* related to *Eospilarctia yuennanica yuennanica* (Daniel, 1943) based on male genital characters

**Table:** 

1	Valva serrate at apex	2
–	Valva rounded or with one excavation at apex (Plate 2: figs 7, 8, 10, 11)	3
2	Apical diverticulum of vesica near long as wide; subbasal diverticulum straight (Plate 2: fig. 3)	*Eospilarctia yuennanica yuennanica*
–	Apical diverticulum of vesica narrow, about three time long as wide; subbasal diverticulum foot-shaped (Plate 2: fig. 6)	*Eospilarctia yuennanica fansipana* ssp. n.
3	Uncus two times longer than width (Plate 2: figs 7, 8; Plate 6: figs 1, 2)	*Eospilarctia maciai* sp. n.
–	Uncus same length as width (Plate 2: figs 10, 11)	4
4	Apex of valva deeply excavated; uncus cruciform Dubatolov et al. 2008, fig. 21, p. 136	*Eospilarctia guangdonga*
–	Apex of valva diagonally cuted, slightly sagged; uncus bulb-shaped (Plate 2: figs 10, 11; Plate 7: figs 1, 2)	*Eospilarctia naumanni* sp. n.

##### Description of new taxa

### Eospilarctia yuennanica fansipana ssp. n.

urn:lsid:zoobank.org:act:C97E1B9C-F2DF-4329-9243-0143C22C99D6

http://species-id.net/wiki/Eospilarctia_yuennanica_fansipana

Plate 1: fig. 3 Plate 2: figs 4–6 Plate 5: figs 1–5

#### Type material.

**Plate 1, figures 1–6.**
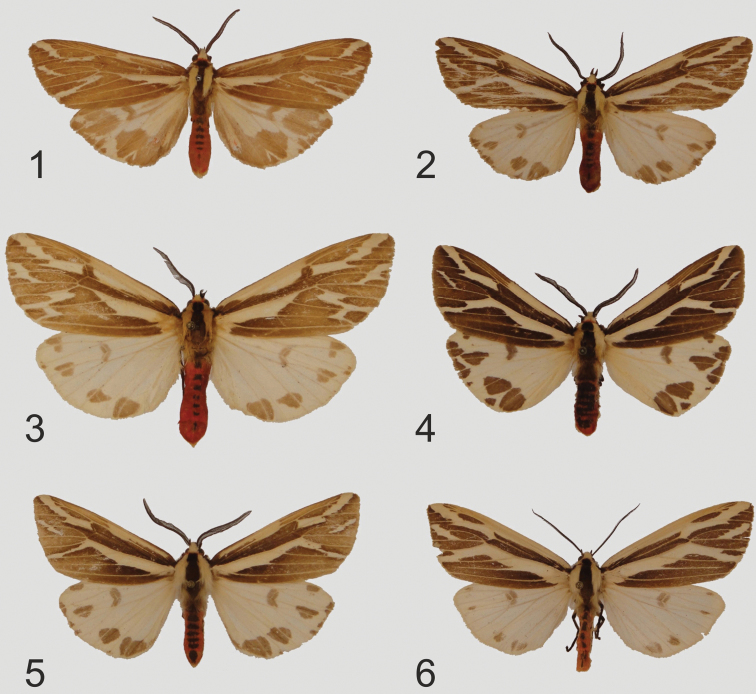
*Eospilarctia* ssp. adults: **1** *Eospilarctia yuennanica yuennanica*, ♂, cotype, China, Yunnan ZSM/MWM **2** *Eospilarctia yuennanica yuennanica*, ♂, China, Yunnan ZSM/MWM **3** *Eospilarctia yuennanica fansipana*, ♂, holotype, Vietnam, Mt. Fan-si-pan ZSM/MWM **4** *Eospilarctia maciai*, ♂, holotype, China, Yunnan ZSM/MWM **5** *Eospilarctia naumanni*, ♂, holotype, Myanmar, Kachin state, ZSM/MWM **6** *Eospilarctia naumanni*, ♀, paratype, Myanmar, Kachin State ZSM/MWM.

**Holotype**: male (Plate 1: fig. 3), northern Vietnam, Mt. Fan-si-pan, 1600-1800 m near Chapa 22°20"N, 103°40"E. Secondary forest, May 1995, leg. local collectors ex coll. A. Schintlmeister (slide No.OP1227), (deposited in ZSM/MWM).

Paratype: male, northern Vietnam, Mt. Fan-si-pan, Chapa, 1700 m NN (22°15"N, 103°46"E), June 1994, leg. Sinjaev & Einheim. Sammler (slide No.OP1226), (deposited in ZSM/MWM).

#### Diagnosis.

The new subspeciesis larger than other *Eospilarctia* species and nominate *Eospilarctia yuennanica yuennanica* (Plates 3, 4). The pale yellow fascia of the forewings are much wider than in the nominate subspecies. Costal fascia of *Eospilarctia yuennanica fansipana* ssp. n. extend to the middle of discal cell and the basal fascia ends in a sharp diagonal tip. *Eospilarctia yuennanica yuennanica* is smaller (cotype forewing 28 mm, wingspan 59mm), forewing markings are similar except that the fascia are much narrower. In the male genitalia the valvae widen without a dorsal excision at apex; juxta with big lateral protrusions, subbasal diverticulum of the vesica has a simple mammilla form; median diverticulum is gently curved, wide.

#### Description.

**Male**: Forewing length of holotype and paratype 33 mm, wingspan 66 mm. Wing pattern typical of genus; forewings’ pale yellow fascia very wide; costal fascia reaching middle of medium cell; basal fascia with sharp diagonal tip.

**Plate 2, figures 1–12.**
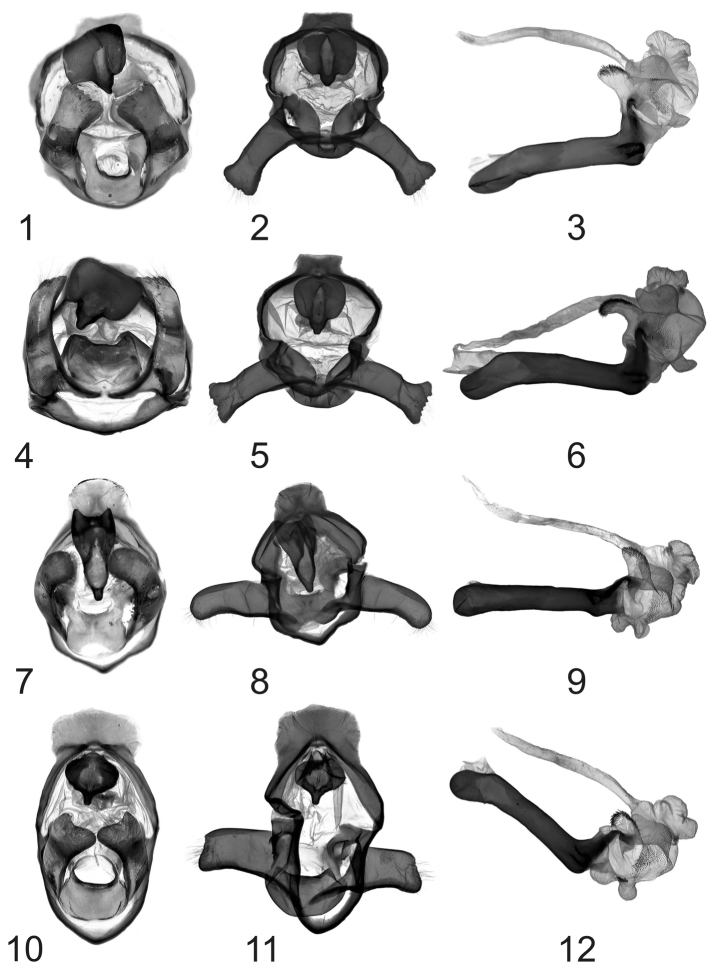
*Eospilarctia* ssp. ♂ genitalia: **1** *Eospilarctia yuennanica yuennanica*, ♂, cotype, clasping apparatus, prep. OP1223 **2** *Eospilarctia yuennanica yuennanica*, ♂, cotype, clasping apparatus with opened valvae **3** *Eospilarctia yuennanica yuennanica*, ♂, cotype, aedeagus **4** *Eospilarctia yuennanica fansipana*, ♂, holotype, clasping apparatus, prep. OP1227 **5** *Eospilarctia yuennanica fansipana*, ♂, holotype, clasping apparatus with opened valvae **6** *Eospilarctia yuennanica fansipana*, ♂, holotype, aedeagus **7** *Eospilarctia maciai*, ♂, holotype, clasping apparatus, prep. OP1225 **8** *Eospilarctia maciai*, ♂, holotype, clasping apparatus with opened valvae **9** *Eospilarctia maciai*, ♂, holotype, aedeagus **10*** E. naumanni*, ♂, holotype, clasping apparatus, prep. OP1228 **11** *Eospilarctia naumanni*, ♂, holotype, clasping apparatus with opened valvae **12** *Eospilarctia naumanni*, ♂, holotype, aedeagus.

**Male genitalia** (Plate 2: figs 4–6): valvae of the same type as the nominate subspecies, but dorsal margin near apex with big excision; oval juxta with wide excision at top, lateral arms very short; aedeagus longer than valvae, gently curved; vesica with footshaped subbasal diverticulum; median diverticulum long, narrow, strongly curved with short spines.

**Female genitalia:** unknown.

**Plate 3, figures 1–5.**
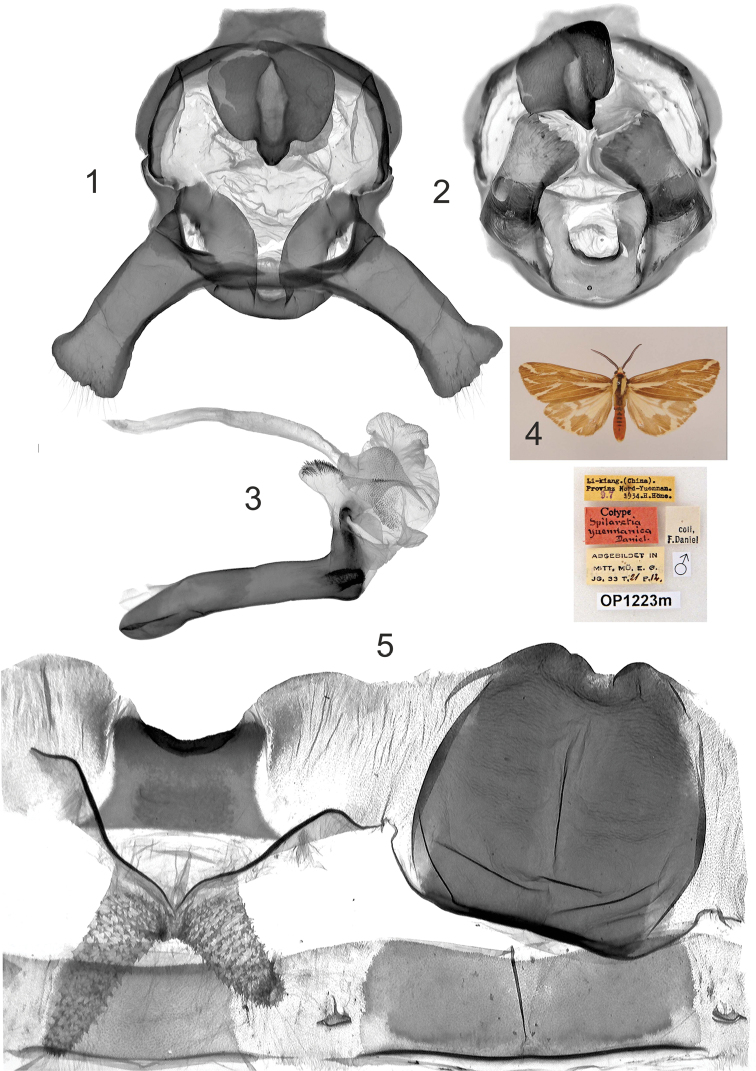
*Eospilarctia yuennanica yuennanica*, ♂, cotype: **1** Clasping apparatus with opened valvae, prep. OP1223 **2** Clasping apparatus **3** Aedeagus **4** Adult 5 8^th^ abdominal segment.

**Plate 4, figures 1–5.**
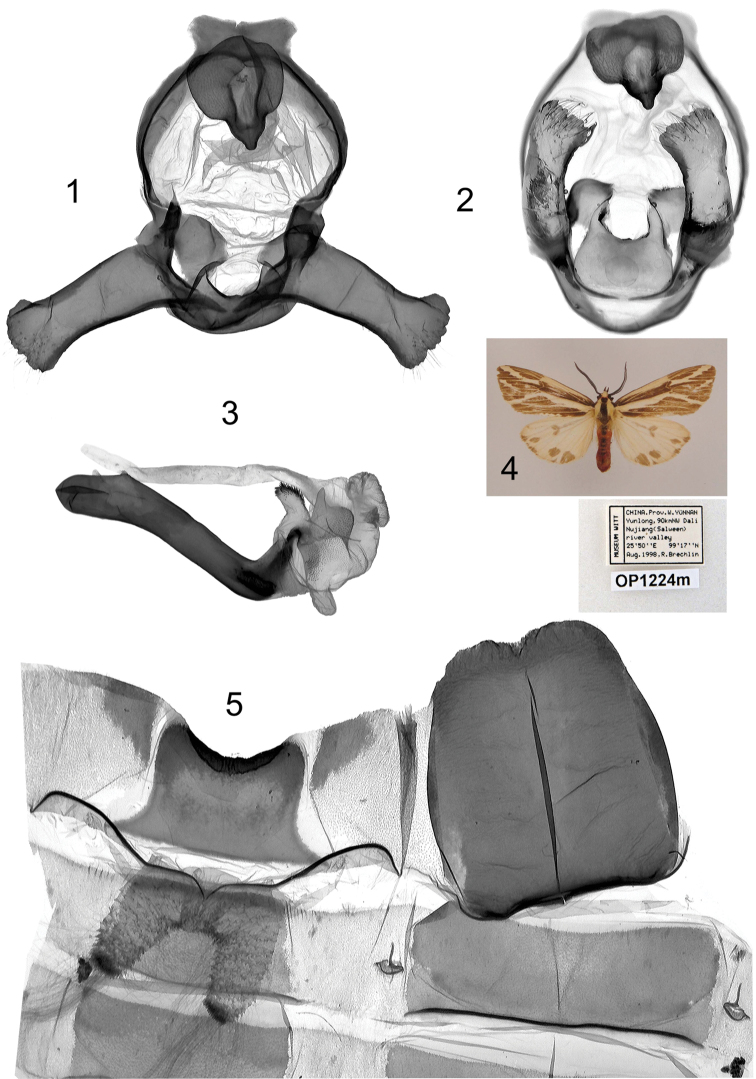
*Eospilarctia yuennanica yuennanica*, ♂: **1** Clasping apparatus with opened valvae, prep. OP1224 **2** Clasping apparatus **3** Aedeagus **4** Adult **5** 8^th^ abdominal segment.

**Plate 5, figures 1–5.**
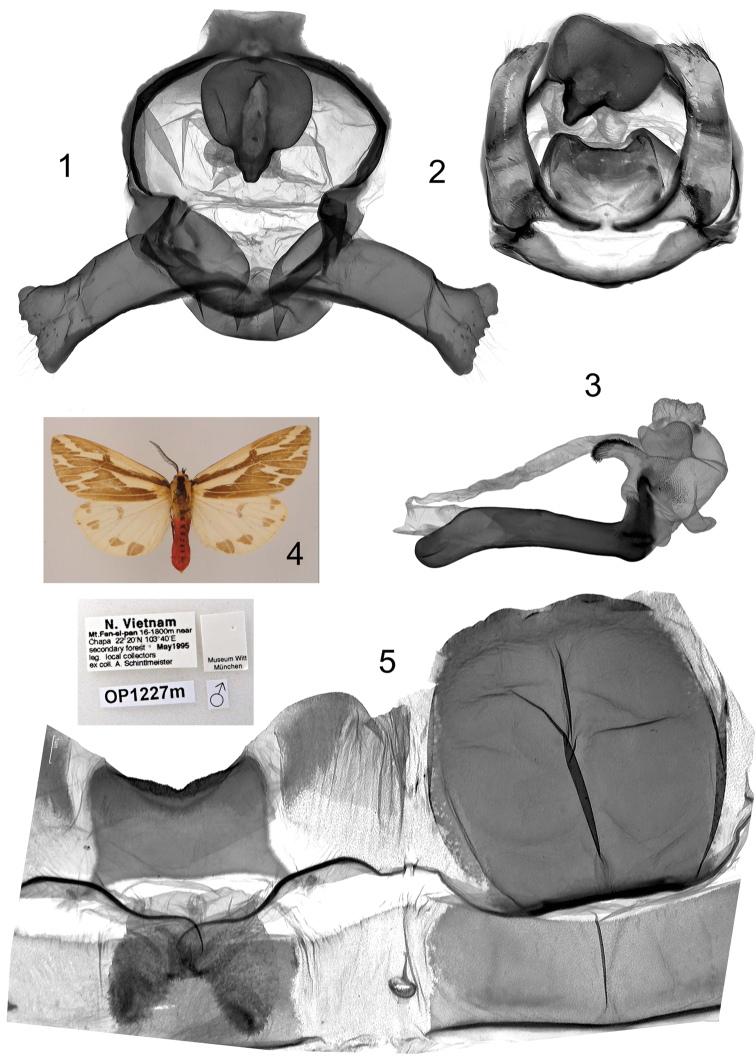
*Eospilarctia yuennanica fansipana*, ♂, holotype: **1** Clasping apparatus with opened valvae, prep. OP1227 **2** Clasping apparatus **3** Aedeagus **4** Adult **5** 8^th^ abdominal segment.

#### Bionomics and distribution.

Known only from the Fan-si-pan mountains in far northern Vietnam. Two male specimens were attracted to light at the end of May at elevations ranging from 1600 to 1800 m.

#### Etymology.

The specific name refers to the type-locality of the new subspecies.

### Eospilarctia maciai sp. n.

urn:lsid:zoobank.org:act:76553FDF-F6BC-4FC8-9BBC-92843706BAED

http://species-id.net/wiki/Eospilarctia_maciai

Plate 1: fig. 4 Plate 2: figs 7–9 Plate 6: figs 1–5

#### Holotype.

**Plate 6, figures 1–5.**
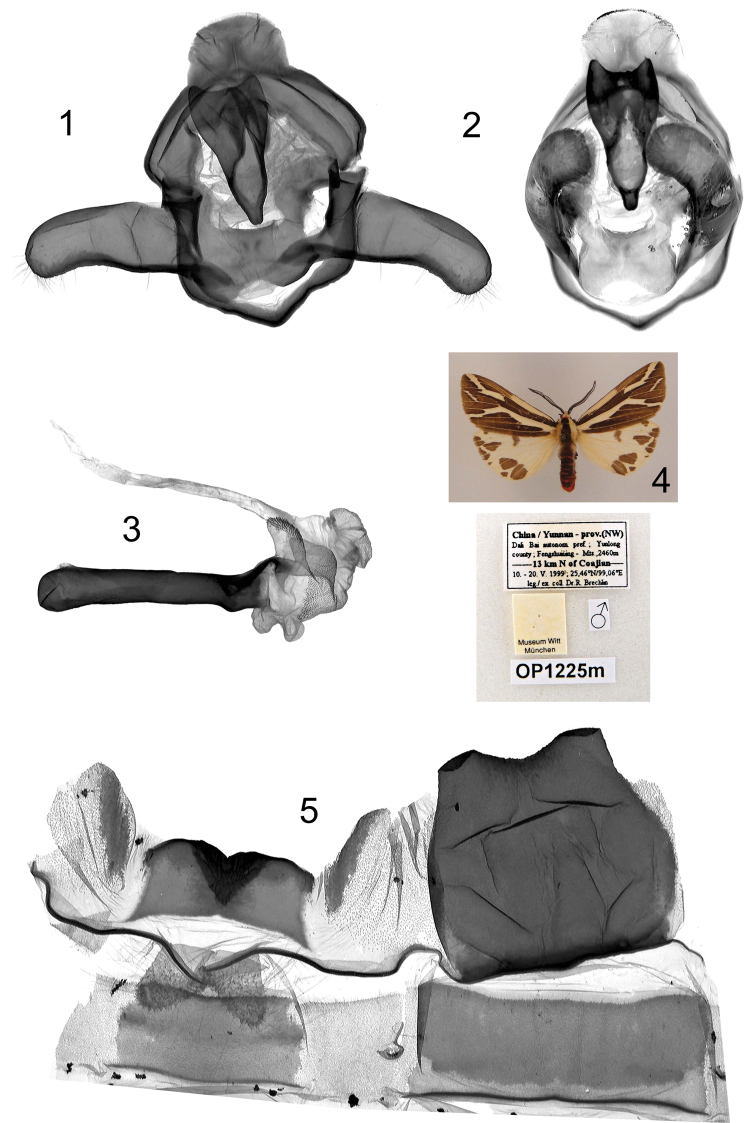
*Eospilarctia maciai*, ♂, holotype: **1** Clasping apparatus with opened valvae, prep. OP1225 **2** Clasping apparatus **3** Aedeagus **4** Adult **5** 8^th^ abdominal segment.

♂ (Plate 1: fig. 4, Plate 6: fig. 4), China, Yunnan prov. Dali Bai autonomy pref., Yulong county, Fengshuiuiny Mts., 2460 m., 13 km. N. of Coajian, 10–20 May 1999, 25°46"N, 99°06"E, leg./ex.coll. Dr R. Brechlin (slide No.OP1225m), (deposited in ZSM/MWM).

#### Diagnosis.

Externallythe new speciesis most similar to the sibling species *Eospilarctia guangdonga* Dubatolov, Kishida & Wang, 2008 status n., *Eospilarctia yuennanica yuennanica* (Daniel, 1943) and *Eospilarctia naumanni* sp. n. In *Eospilarctia guangdonga* (species was recently described and well illustrated (Dubatolov et al 2008)) the wing markings are noticeably paler and in the male genitalia the valvae do not widen at the excavated apex. The forewings of *Eospilarctia yuennanica yuennanica* have more intensive brown markings, with fascia pale white, and the male valvae widen to rough-edged apex. In *Eospilarctia naumanni*, the costal fascia from the forewing base do not extend to apex of medial cell. In the male genitalia the valvae are almost the same width as length, slightly curved at the middle and wide at tip; saccus massive, slightly narrowing with a blunt tip; juxta “X” shaped, top and bottom deep, juxta lateral sides with weak excavations; aedeagus straight, longer than valva, with visible bulge at ventral tip.

#### Description.

**Male**: Forewing length of holotype 26 mm, wingspan 50 mm; antennae strongly bipectinate; ground color of forewings dark blackish brown, veins yellow, wing pattern typical of genus. Costal fascia from base not extending to M cell apex. Terminal streak from M2 straight, direct to termen at 45 degree angle crossing vein M1, narrow, with even edges. Hindwings whitish yellow, with brown patches, ventral pattern and color of wings similar to dorsal.

**Male genitalia** (Plate 2: figs 7–9, Plate 6: figs 1–3): Uncus severely narrowing to blunt tip; valvae almost the same width as length, slightly curved medially to the wide, oval apex; tegumen wide, narrowing; saccus massive, slightly narrowing to blunt tip; juxta wide at top and bottom deep, sides slightly excavated; aedeagus straight, longer than valvae, with ventral bulge at tip; vesica with two subbasal diverticula.

**Female :** unknown.

#### Bionomics and distribution.

Known only from the Fengshuiuiny Shan Mountains in Yunnan province in southwest China, *Eospilarctia maciai* sp.n. is likely endemic to Yunnan, and the nearby regions of Myanmar. The single male specimen was attracted to light in mid-May at an elevation of 2500 m.

#### Etymology.

The new species is named after Mr Ramon Macià Vilà (Barcelona, Spain), the famous Iberian Peninsula’s Arctiidae specialist.

### Eospilarctia naumanni sp. n.

urn:lsid:zoobank.org:act:08D02B41-D6F3-4872-B2B2-1C9CB31A86D0

http://species-id.net/wiki/Eospilarctia_naumanni

Plate 1: fig. 5, 6 Plate 2: figs 10–12 Plate 7: figs 1–5 Plate 8: figs 1–5 Plate 9: figs 1, 2

#### Type material.

**Plate 7, figures 1–5.**
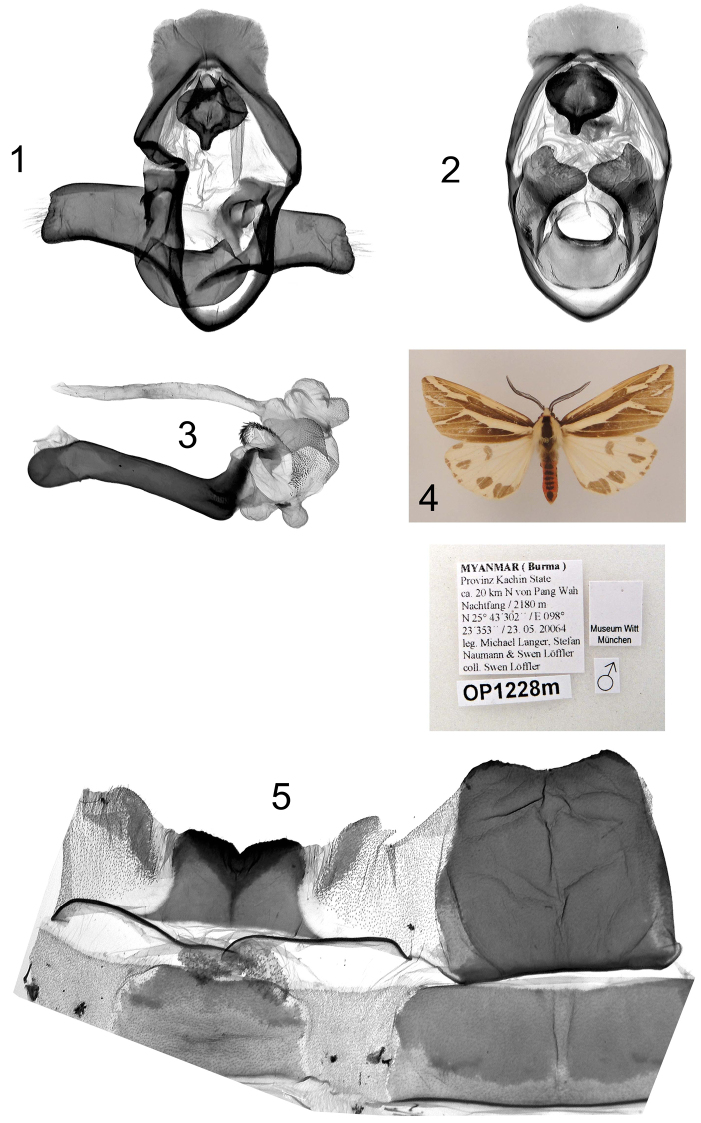
*Eospilarctia naumanni*, ♂, holotype: **1** Clasping apparatus with opened valvae, prep. OP1228 **2** Clasping apparatus **3** Aedeagus **4** Adult **5** 8^th^ abdominal segment.

**Holotype**: ♂ (Plate 1: fig. 5, Plate 7: fig. 4), Northeast Myanmar (Burma), Kachin state, road Chibwe - Pang Wah, 2 km N branch to Kanphant, 2180 m, 25.4251°N, 98.5431°E, 23 May, 2006 at light, leg. Stefan Naumann, Michael Langer, & Swen Löffler, ex. coll. Swen Löffler (slide No.OP1228) (deposited in ZSM/MWM).

**Plate 8, figures 1–5.**
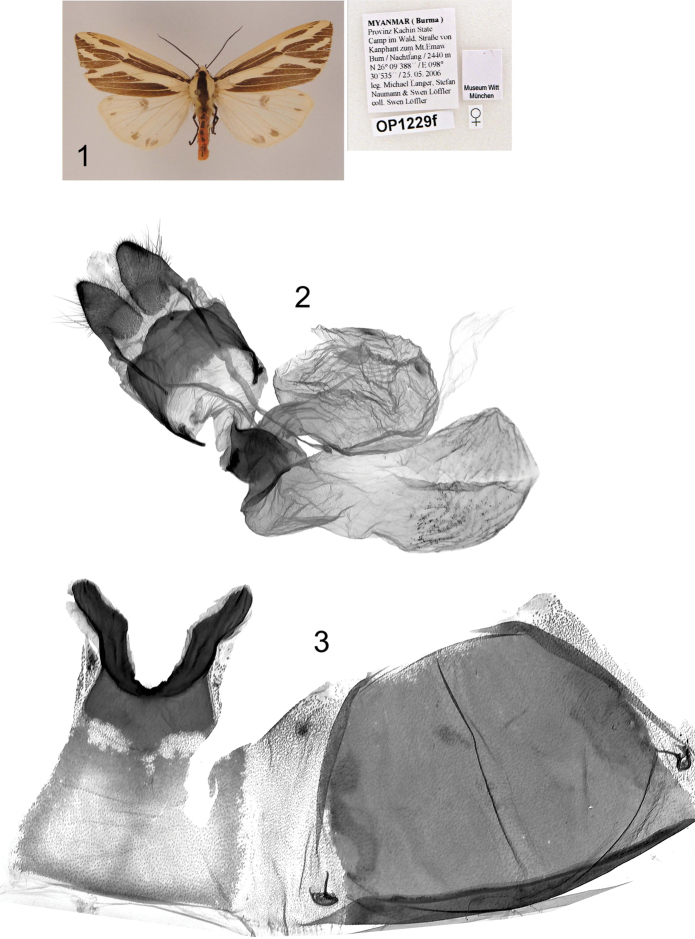
*Eospilarctia naumanni*, ♀, paratype: **1** Adult **2** Ductus bursa, prep. OP1229 **3** 7^th^ abdominal segment.

Paratype: ♀ (Plate 1: fig. 6, Plate 8: fig. 1), Northeast Myanmar, road Kanphant – Mt. Inwa Bum, forest camp, 2440 m, 26.1608°N, 98.35149°E, 25 May, 2006, leg. S. Naumann, M. Langer, & S. Löffler, ex. coll. Swen Löffler (slide No.OP1229f), (pers. comm. S. Naumann) (deposited in ZSM/MWM).

#### Diagnosis.

Externallythe new speciesis most similar to sibling species *Eospilarctia guangdonga* Dubatolov, Kishida & Wang, 2008, *Eospilarctia yuennanica* (Daniel, 1943) and*Eospilarctia maciai* sp. n. In *Eospilarctia guangdonga* thewing markings are noticeably paler and in its male genitalia the valvae are not widening at the apex, here with a noticeable excavation. *Eospilarctia yuennanica* forewings are intense brown with pale white markings, in male genitalia the valvae are widening with cut and rough apex. In *Eospilarctia maciai*, the forewingterminal streak from M_2_ is straight, extending to termen at about a 45° angle, crossing vein M_1_, narrow, with even edges. The costal fascia does not extend to apex of the medial cell. In the male genitalia valvae almost the same width as long, in middle slightly curved, tip of valva wide, oval, tegumen wide narrowing, saccus with well visible blunt tip, juxta “X” form top and bottom wide deep, sides slightly excavated; aedeagus straight longer than valva, at the tip in ventral side with visible bulge.

#### Description.

**Male**: Forewing length of holotype 26 mm, wingspan 54 mm; antennae strongly bipectinate; forewing veins yellow on brown background; wing pattern typical of genus. Yellow costal band extends from forewing basis to apical streak. Terminal streak from M_2_ straight, direct to termen 35° angle, cross vein M1, narrow, with rough edges. Hindwing white yellow, with brown spots. Ventral wing pattern and color identical to upper side.

**Female.** Forewing length of paratype 27 mm; wingspan 55 mm; antennae weakly bipectinate; patches on hindwings very small; other morphological feature as in male.

**Male genitalia** (Plate 2: figs 10–12, Plate 7: figs 1–3). Uncus bulbous; valvae short, almost the same width as length; slight curvature in mid-ventral portion of valve with tip transversely cut; tegumen narrow; saccus slightly narrowing without visible blunt tip; juxta wide, rounded, top deeply excavated; aedeagus straight, longer than valva, at the tip curved nearly 90°, vesica with two subbasal diverticula, one of them twice as wide as the other; median diverticulum short with short spines.

**Female genitalia.** (Plate 8: figs 2, 3). Papilla analis massive; apophysis posterioris about two and half times shorter than apophysis anterioris; ductus short; bursa bisaccate, rounded; eighth abdominal sternum with lateral sclerites; dorsum sclerotised, simple.

#### Bionomics and distribution.

**Plate 9, figures 1, 2.**
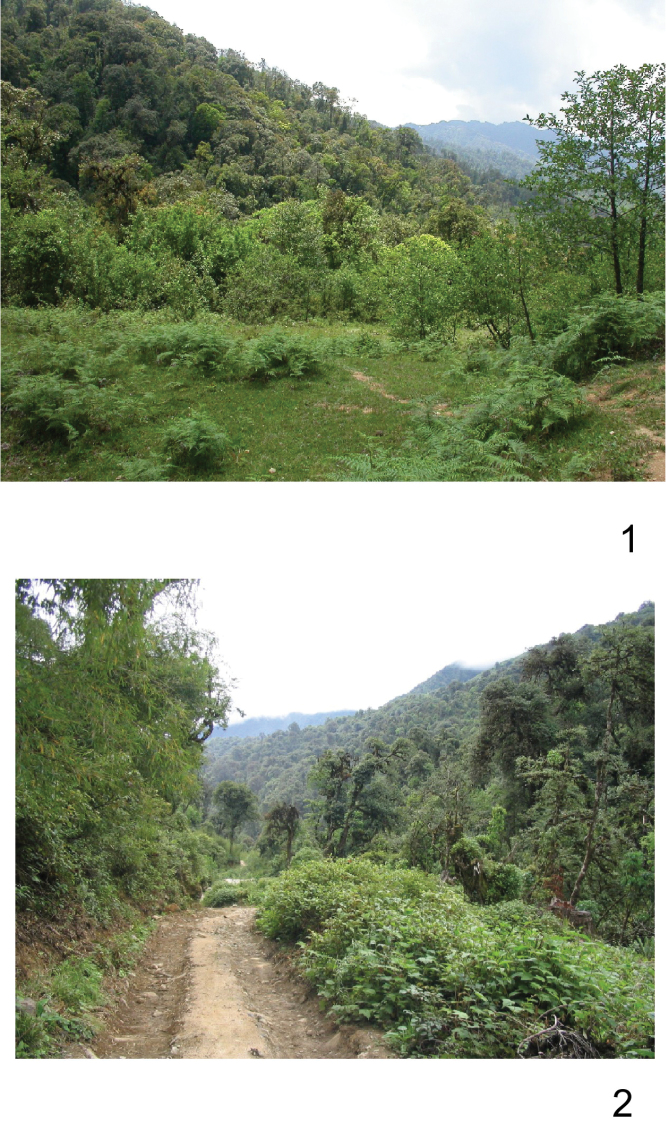
*Eospilarctia naumanni*, type locality’s: **1** Myanmar NE, Kachin state, road Chibwe – Pan Wah, 2 km N branch to Kanphant **2** Myanmar NE, Kachin state, road Kanphant – Mt. Inwa Bum.

*Eospilarctia naumanni*sp.n.is known only from the Kachin region of northern Myanmar (Plate 9: figs 1, 2). The new species was collected in late May at elevations of 2200 to 3000 m in mountainous virgin mixed forest, with swampy and mossy meadows. The habitat dominated by various species of *Alnus*, *Prunus*, *Quercus*, *Rhododendron*, *Abies*, different species of small bamboos and other smaller shrubs and ferns. Specimens of the new species were attracted to light (pers. comm. S. Naumann).

#### Etymology.

The new species is named after Mr Stefan Naumann (Berlin, Germany), a renowned Saturniidae specialist.

Dubatolov, Kishida and Wang (2008) described *Eospilarctia yuennanica guangdonga* as a new subspecies and included imago and male genitalia images, however we rise this taxa to species status, *Eospilarctia guangdonga* stat. nov. The *Eospilarctia guangdonga* forewing length is 31 mm compared to 28mm for *Eospilarctia yuennanica yuennanica*. In *Eospilarctia guangdonga* male genitalia the uncus is cruciform, valvae are not dilatable, tips of valvae are excavated, the juxta has short lateral protrusions, and aedeagus is shorter and stouter.In *Eospilarctia yuennanica yuennanica* male genitalia the valvae widen without excision at dorsal side of the apex, the juxta have bigger lateral extensions, and aedeagus is longer and thinner.

## Checklist of the genus *Eospilarctia*

Species distributions are given in Dubatolov (2010).

***Eospilarctia chuanxina* (Fang, 1982)**

Holotypus: Institute of Zoology, Academic Sinica (Beijing, China). Type locality: Sichuan. Distribution: China: Sichuan.

***Eospilarctia fangchenglaiae* Dubatolov, Kishida & Wang, 2008**

Holotypus: Siberian Zoological Museum (Novosibirsk, Russia). Type locality: “Vietnam, cao Bang, Mt. Pia Oac, h=1700 m”. Distribution: Northern Vietnam (Lao cai, Cao Bang); China: Sichuan, Yunnan, Guangdong, Zhejian, Jiangxi, Shaanxi, Hubei, Hunan.

***Eospilarctia formosana* (Rothschild, 1933)**

Holotypus: Natural History Museum (London, UK). Type locality: “Rantaizan, Arizan”[Taiwan]. Distribution: Taiwan.

***Eospilarctia guangdonga* Dubatolov, Kishida & Wang, 2008, stat. n.**

Holotypus: South China Agricultural University (Guangzhou, China). Type locality: Guangdong, China. Distribution: China: Guangdong.

***Eospilarctia huangshanensis* Fang, 2000**

Holotypus: Institute of Zoology, Academic Sinica (Beijing, China). Type locality: “Anhui, Huangshan” [China]. Distribution: China: Anhui, Huangshan.

***Eospilarctia lewisii* (Butler, 1885)**

Holotypus: Natural History Museum (London, UK). Type locality: [Japan]. Distribution: Japan: Honshu, Shikoku, Kyushu, Tsushima.

***Eospilarctia maciai* sp. n.**

Holotypus: Zoologische Staatssammlung der Bayerischen Staaten (Munich, Germany)/Museum of Thomas Witt. Type locality: Yunnan prov. Dali Bai autonomy pref., Yulong county, Fengshuiuiny Mts., 2460 m (China). Distribution:China: Yunnan.

***Eospilarctia naumanni* sp. n.**

Holotypus: Zoologische Staatssammlung der Bayerischen Staaten (Munich, Germany)/Museum of Thomas Witt. Type locality: Northeast Myanmar (Burma), Kachin state, road Chibwe - Pang Wah. Distribution: Myanmar: Kachin.

***Eospilarctia nehallenia nehallenia* (Oberthür, 1911)**

Holotypus: Natural History Museum (London, UK). Type locality: “Tâ-tsien-Lou” [China: Sichuan]. Distribution: China: Sichuan, Shaanxi, Yunnan.

***Eospilarctia nehallenia baibarensis* (Matsumura, 1930)**

Holotypus: National Science Museum (Tokyo, Japan). Type locality: “Formosa at Baibara near Horisha” [Taiwan]. Distribution: Taiwan.

***Eospilarctia neurographa* (Hampson, 1909)**

Holotypus: Natural History Museum (London, UK). Type locality: “Formosa, Kagi distr.” [Taiwan]. Distribution: Taiwan.

***Eospilarctia pauper* (Oberthür, 1911)**

Holotypus: Natural History Museum (London, UK). Type locality: “Siao-Lou” [China: Sichuan]. Distribution: China: Sichuan, Yunnan.

***Eospilarctia taliensis* (Rothschild, 1933)**

= *jordansi* (Daniel, 1943)

Cotypus: Zoologische Staatssammlung der Bayerischen Staaten (Munich, Germany)/Museum of Thomas Witt. Type locality: “Tali Haut, Yunnan” [China]. Distribution: China: Yunnan, Sichuan (?), Shaanxi.

***Eospilarctia yuennanica yuennanica* (Daniel, 1943)**

Cotypus: Zoologische Staatssammlung der Bayerischen Staaten (Munich, Germany)/Museum of Thomas Witt. Type locality: “Li-kiang (Nord Yuennan)” [China]. Distribution: China: Sichuan, Yunnan.

***Eospilarctia yuennanica fansipana* ssp.n**

Holotypus: Zoologische Staatssammlung der Bayerischen Staaten (Munich, Germany)/Museum of Thomas Witt. Type locality: northern Vietnam, Mt. Fan-si-pan, 1600–1800 m near Chapa. Distribution: Vietnam: Chapa, Mt. Fan-si-pan.

## Supplementary Material

XML Treatment for Eospilarctia

XML Treatment for Eospilarctia yuennanica fansipana

XML Treatment for Eospilarctia maciai

XML Treatment for Eospilarctia naumanni
